# Association of Polycyclic Aromatic Hydrocarbons Urine Metabolites with Type 1 Diabetes

**DOI:** 10.1155/2023/6692810

**Published:** 2023-06-23

**Authors:** Roya Kelishadi, Silva Hovsepian, Mohammad Mehdi Amin, Nafiseh Mozafarian, Sara Sedaghat, Mahin Hashemipour

**Affiliations:** ^1^Child Growth and Development Research Center, Research Institute for Primordial Prevention of Non-Communicable Disease, Isfahan University of Medical Sciences, Isfahan, Iran; ^2^Metabolic Liver Disease Research Center, Isfahan University of Medical Sciences, Isfahan, Iran; ^3^Environment Research Center, Research Institute for Primordial Prevention of Non-Communicable Disease, Isfahan University of Medical Sciences, Isfahan, Iran; ^4^Gabric Diabetes Education Association, Tehran, Iran

## Abstract

**Purpose:**

Polycyclic aromatic hydrocarbons (PAHs) are believed to be a possible factor in the development of cancer, ischemic heart disease, obesity, and cardiovascular disease. The objective of this study was to explore the association between certain metabolites of urinary PAH and type 1 diabetes (T1D).

**Methods:**

In Isfahan City, a case-control study was carried out involving 147 T1D patients and an equal number of healthy individuals. The study measured the levels of urinary metabolites of PAHs, specifically 1-hydroxynaphthalene, 2-hydroxynaphthalene, and 9-hydroxyphenanthrene, in both the case and control groups. The levels of these metabolites were then compared between the two groups to assess any potential association between the biomarkers and T1D.

**Results:**

The mean (SD) age of participants in the case and control groups was 8.4 (3.7) and 8.6 (3.7) years old, respectively, (*P* > 0.05). In terms of gender distribution, 49.7% and 46% of participants in the case and control groups were girls, respectively (*P* > 0.05). Geometric mean (95% CI) concentrations were: 36.3 (31.4-42) *μ*g/g creatinine for 1-hydroxynaphthalene, 29.4 (25.6-33.8) *μ*g/g creatinine for 2-hydroxynaphthalene, and 72.26 (63.3-82.5) *μ*g/g creatinine for NAP metabolites. After controlling for variables such as the child's age, gender, maternal and paternal education, duration of breastfeeding, exposure to household passive smoking, formula feeding, cow's milk consumption, body mass index (BMI), and five dietary patterns, it was observed that individuals in the highest quartile of 2-hydroxynaphthalene and NAP metabolites had a significantly greater odd ratio for diabetes compared to those in the lowest quartile (*P* < 0.05).

**Conclusion:**

Based on the findings of this study, it is suggested that exposure to PAH might be linked to an increased risk of T1D in children and adolescents. To clarify a potential causal relationship related to these findings, further prospective studies are needed.

## 1. Introduction

T1D is a prevalent chronic endocrine condition among children and adolescents globally. [1]. Although its prevalence is lower than that of type 2 diabetes with recent evidence of its increasing trend, it is becoming one of the emerging health problems in pediatric endocrinology [2, 3]. Over the past 3 decades, an upward trend in T1D has been reported to be 3-4% [4].

Although environmental and genetic factors play an important role in the pathogenesis of T1D, evidence indicates that the recent upward trend may be mainly due to environmental factors [5, 6].

However, recent studies propose that the rising incidence of T1D could be attributed to a category of environmental factors known as endocrine-disrupting chemicals (EDCs), such as phthalates, bisphenol A, polycyclic aromatic hydrocarbons (PAHs), pesticides, and flame retardants [6, 7].

Recognizing the role of EDCs in the development of T1D is deemed a significant matter due to its potential implications for the design of prevention strategies [8]. The role of the mentioned EDCs has been studied in many recent studies [9, 10]. One of the important EDCs is PAH. Most of the available literature regarding the mechanisms and association of PAH and its metabolites with T1D has been obtained from experimental studies [11] [12]. Accordingly, systematic inflammation or oxidative stress induced by PAH can lead to the disruption of insulin signaling pathways and *β*-cell dysfunction. Air pollution is the primary cause of elevated exposure to PAHs [11, 12].

The results of a systematic review indicate that children and adolescents are more susceptible to exposure to PAHs and related complications than non-occupational exposures in adults [13].

Although numerous epidemiological studies have proposed a correlation between exposure to PAHs and diabetes [11, 12, 14, 15], there are no studies in children and specifically for T1D. Therefore, given the importance of identifying associations between environmental factors and T1D to explain the growing trend and the evolution of prevention strategies, we sought to identify the association between exposure to PAHs as measured by its urine concentration and T1D in Isfahan, Iran.

## 2. Methods

Between 2020 and 2021, we conducted a case-control study on individuals aged 5-18 years old in Isfahan City to compare T1D patients and healthy controls. Newly diagnosed T1D patients were selected as the case group from various private pediatric endocrinologists' clinics and the diabetes clinic of Imam Hossein Children's Hospital. The control group was matched to the case group based on their place of residence. The control group consisted of siblings or cousins or neighbors of the children with T1D. Before conducting the research, the ethics committee of Isfahan University of Medical Sciences approved the study with the ethics code IR.MUI.REC.1396.1.082 and research project number 196082. The selected participants and their parents provided written informed consent prior to the research.

Information on demographic characteristics, socioeconomic status, area of residence, participants' diet, nutritional type in early life, and passive smoking was collected through a validated questionnaire. The study population underwent physical measurements of weight and height by a qualified nurse using reliable instruments. The body mass index (BMI) was then calculated as the ratio of weight in kilograms to the square of height in meters. Additionally, the levels of urinary metabolites of PAH, specifically 1-hydroxynaphthalene, 2-hydroxynaphthalene, and 9-hydroxyphenanthrene, were quantified from urine samples obtained from the study participants, and levels of different metabolites were compared in case and control groups.

### 2.1. Laboratory Analysis

Spot urine samples were collected from both groups, and the concentrations of PAH metabolites were measured using gas chromatography coupled with high-resolution mass spectrometry. Specifically, they measured 1-hydroxynaphthalene, 2-hydroxynaphthalene, and 9-hydroxyphenanthrene, and the method was described in detail in previous studies [16, 17].

The limits of detection (LOD) for each metabolite were as follows: 0.27 for 1-hydroxy naphthalene, 0.11 for 2-hydroxynaphthalene, and 0.14 for 9-hydroxyphenanthrene. If a concentration was below the LOD, the researchers substituted a value equal to half of the LOD [18]. The statistical analysis also involved adjusting the PAH metabolite concentrations for urinary creatinine concentrations. Also, another variable was created: the sum of 1-hydroxy naphthalene and 2-hydroxynaphthalene as NAP metabolites.

### 2.2. Dietary Patterns

Based on the participants' eating habits and their consumption of different food groups, principal component analysis (PCA) was used to establish dietary patterns.

According to the PCA method with a varimax rotation, five dietary patterns were identified among the participants: (1) an unhealthy snacking diet that contains a lot of salty snacks and chips; (2) a western pattern that was rich in mayonnaise, sausages, fried burgers, fast food, and soft drinks; (3) high-protein diet which was rich in fried white and red meat; (4) Seafood diet which was rich in fried and grilled fish; and (5) canned foods which was high intake of canned foods. The dietary patterns were then divided into tertiles, with the first tertile representing low consumption, the second tertile representing moderate consumption, and the third tertile representing high consumption levels.

### 2.3. Statistical Analysis

Statistical analysis was performed using STATA 10 software (StataCorp, College Station, Texas, USA). The data in the study were reported as mean (SD) and median (25th-75th percentile) for continuous variables and frequency (%) for categorical variables. To determine if there were any differences in the concentration of urinary PAH metabolites between children with and without T1D, the Mann–Whitney test was conducted. The researchers categorized the concentrations of urinary PAH metabolites into quartiles to estimate the association between these biomarkers and T1D. Multiple logistic regression analysis was used, while taking into account several confounding variables such as age, sex, maternal and paternal education, duration of breastfeeding, exposure to household passive smoking, formula feeding, breast milk and cow's milk consumption, BMI, and five dietary patterns.

## 3. Results

In this case-control study, 147 T1D patients and 147 healthy children and adolescents were included. Baseline characteristics of the participants are shown in Table 1.

**Figure 1:**
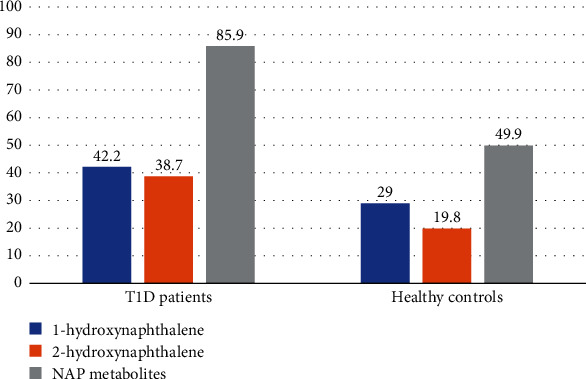
Median urinary PAHs metabolites in patients with type 1 diabetes and healthy control groups.

The duration of breastfeeding was significantly longer in healthy participants (*P* = 0.01).  Figure 1 displays the mean urinary PAH metabolite levels for both the T1D patient group and the control group. The levels of metabolites were found to be significantly higher in T1D patients than in the control group (*P* < 0.05).  Table 2 presents the geometric mean (95% CI) and range of urinary PAH metabolites that were adjusted for urinary creatinine. Approximately 93% of the participants had concentrations below the LOD of the 9-phenanthrene metabolite. Therefore, this metabolite was excluded from our analysis. According to the results, individuals with T1D had significantly higher concentrations of 1-hydroxynaphthalene, 2-hydroxynaphthalene, and NAP metabolites compared to those without T1D.

Table 3 displays a multivariable logistic regression model that was used to assess the correlation between urinary PAH metabolite concentrations and T1D. The results showed a higher odd ratio (OR) of T1D in participants who were in quartiles 3 and 4 of 2-hydroxy naphthalene and NAP metabolites, compared to those in the lowest quartile (*P* < 0.05). However, no significant association was observed between 1-hydroxynaphthalene and T1D.

Table 4 presents the association between PAH metabolites and T1D in girls and boys.

In boys, the highest OR of T1D was found for NAP, and in girls, the highest OR was found for 2-hydroxynaphthalene.

## 4. Discussion

The aim of this study was to investigate the potential link between T1D and levels of PAH metabolites in urine. Our findings indicated a significant association between T1D, 2-hydroxynaphthalene, and NAP metabolites.

Assessing the involvement of endocrine-disrupting chemicals (EDCs) in the development of T1D is a challenging issue. Based on current evidence, EDCs could have direct and indirect toxic effects on insulin production. Directly, they could impair the function of beta cells by changing hormonal levels, immunomodulatory effects, or by changing the microbiota and intestinal permeability. Indirectly, they can alter gene expression due to chemical-induced epigenetic changes [19].

PAHs are among the most important EDCs with highly toxic effects associated with many reported diseases. It may have immunotoxic, genotoxic, reproductive and developmental toxicity, and carcinogenic effects [19, 20].

The sources of exposure to PAHs in humans are smoking (both active and passive), occupation, dietary sources, water, and mainly air pollution [19, 20].

Children are considered a high-risk population for PAH exposure through the air, food, and soil. Children's behavioral patterns, including greater inhalation and swallowing patterns, smaller body size, and less developed detoxification systems, may be responsible for the higher risk of exposure [21].

Furthermore, the findings of a study carried out in our locality indicated a significant association between air pollution and urinary PAH levels in children [22].

Currently, there are no studies that have investigated the correlation and mechanisms between PAH exposure and T1D. However, in vitro studies have suggested that exposure to PAHs through air pollution can potentially cause the dysfunction of regulatory T cells (Treg), which may play a role in the onset of T1D [19, 23].

A systematic review and meta-analysis conducted recently have revealed a connection between PAH and diabetes, with the majority of the studies indicating a correlation with type 2 diabetes (T2D). Studies including two types of diabetes have not demonstrated an exact difference between them [24].

Most of the identified mechanisms for the diabetogenic effects of PAH are related to insulin resistance, but it has been suggested that from reported biological pathways, oxidative stress is considered an important factor that may lead to beta cell dysfunction [24].

A study conducted by Alshaarawy et al. in the USA analyzed data from the National Health and Nutrition Examination Survey (NHANES) to investigate the link between urinary PAH and diabetes. The study found that there was a significant correlation between diabetes and certain low molecular weight PAH biomarkers, including 1 and 2-hydroxynaphthol and 2-hydroxyphenanthrene. The included participants were 20-65 years old, but they did not demonstrate the type of diabetes, but considering the age of the participants, they suggested that most of the participants had T2D [25]. Similarly, Nam and Kim recently investigated the association between diabetes and T2D in Korea using data from participants in cycle 2 (2012-2014) of the Korean National Environmental Health Survey (KoNEHS). The study revealed a significant correlation between diabetes and urinary levels of 2-NAP and 2-OHFlu. However, it had a limitation as the patients were not categorized by type of diabetes. [14].

In this study, the urinary level of PAH metabolites was significantly higher in patients than in healthy ones, but a further analysis indicated that the association between PAH and T1D was mainly due to the level of 2-hydroxynaphthalene and NAP.

In the present study, there was no significant association between 1-hydroxynaphthalene and T1D. Our findings are consistent with the results of the recent meta-analysis by Khosravipour et al. Nor did they find such an association. Based on their suggestion, although 1-hydroxy naphthalene is considered to be one of the most important metabolites of PAHs, it is mainly related to low exposure [24], so there is no definitive evidence regarding the diagnostic value of urinary 1-hydroxy naphthalene.

Similar to Nam and Kim's study, we evaluated the association of T1D with urinary creatinine-adjusted PAH levels [14].

Several studies have suggested that males and urban populations tend to have higher levels of urinary PAH concentrations [26, 27]. It may be due to males having more outdoor activities and therefore more exposure. Recently, Costanzo et al. reported that sex differences in various aspects of health-related research are due to metabolic alterations affecting proteins, genomes, and transcription factors [28].

This study found that the association between T1D and PAH metabolites differed between males and females. In male patients, there was an association between NAP and T1D, while in females, the association was with 2-hydroxynaphthalene. The difference could be explained by the difference in exposure between the sexes to different PAH metabolites.

The main limitation of this study was the small sample size of the participants. The strength of our study was that the association was analyzed after adjusting for confounders, and we used the quartiles of the level of the metabolites. As far as we know, this study is the first to investigate the possible link between exposure to PAHs and T1D in children.

## 5. Conclusion

This study found that the levels of 2-hydroxynaphthalene and NAP metabolites in urine were linked to T1D in Iranian children. Our findings as background information will help to investigate the mechanisms of the EDCs in the development of T1D. In addition, we recommend that further studies be designed to evaluate the most important sources of PAH exposure in our community and the additive effects of other EDCs on PAH in the pathogenesis of T1D.

## Figures and Tables

**Table 1:** Characteristics of the children with type 1 diabetes and control group.

Characteristics	Controls *n* = 147	Children with type 1 diabetes *n* = 147	*P* value
Age (year)^€^	8.6 (3.7)	8.4 (3.7)	0.668
Weight (kg)^€^	32.2 (14.6)	29.4 (13.8)	0.111
BMI (kg/m2)^€^	17.6 (4.6)	16.4 (4.1)	0.033
Gender (girls/boys)	69 (46.9)/78 (53.1)	73 (49.7)/74 (50.3)	0.641
Living area			
Rural	36 (24.5)	38 (25.9)	0.788
Urban	111 (75.5)	109 (74.1)	
Paternal education			
Illiterate/elementary	22 (15)	27 (18.5)	
Secondary school/high school	102 (69.4)	93 (63.7)	0.575
Academic	23 (15.6)	26 (17.8)	
Maternal education			
Illiterate/elementary	24 (16.3)	23 (15.8)	
Secondary school/high school	103 (70.1)	102 (69.9)	0.977
Academic	20 (13.6)	21 (14.4)	
Breastfeeding duration			
<6 months	5 (3.5)	18 (12.5)	
6 to 12 months	8 (5.6)	5 (3.5)	0.016
>12 months	129 (90.8)	121 (84)	
Familiar history of diabetes	19 (16.1)	15 (11.8)	0.332
Passive smoking at home(yes)	50 (35.5)	62 (43.4)	0.173
Formula feeding(yes)	19 (12.9)	30 (20.5)	0.080
Breastfeeding(yes)	144 (98)	136 (93.2)	0.046
Cow milk(yes)	3 (2)	9 (6.2)	0.073

€Mean (SD).

**Table 2:** Exposure distribution of urinary metabolites of polycyclic aromatic hydrocarbons corrected for urinary creatinine.

PAH metabolites	Range of PAH per gram of creatinine (*μ*g/g cratinine)				Geometric mean (95% CI)
	Quartile 1	Quartile 2	Quartile 3	Quartile 4	
1-Hydroxynaphthalene	0.86-15.85	15.86-34.17	34.18-81.94	81.95-1776.6	36.3(31.4-42)
2-Hydroxynaphthalene	0.62-13.5	13.5-27.6	27.6-66.95	66.96-763.96	29.4(25.6-33.8)
NAP metabolites	5.12-31.4	31.5-66.7	66.8-158.5	158.6-2181	72.26(63.3-82.5)

NAP metabolites; the sum of 1-hydroxynaphthalene and 2-hydroxynaphthalene (creatinine-corrected concentration (*μ*g/g creatinine)).

**Table 3:** Association of urinary levels of PAHs metabolites with type 1 diabetes.

Metabolites (*μ*g/g creatinine)		Crude models		Adjusted model^∗^		Adjusted model^∗∗^	
		OR	*P* value	OR	*P* value	OR	*P* value
1-Hydroxynaphthalene	Quartile 1	Ref		Ref		Ref	
	Quartile 2	0.85	0.617	0.85	0.659	1.4	0.491
	Quartile 3	1.25	0.508	1.33	0.419	1.7	0.249
	Quartile 4	1.64	0.137	1.53	0.222	2.2	0.098

2-Hydroxynaphthalene	Quartile 1	Ref		Ref		Ref	
	Quartile 2	1.93	0.060	1.70	0.167	1.3	0.629
	Quartile 3	4.75	<0.001	3.81	<0.001	4.2	0.002
	Quartile 4	3.98	<0.001	3.45	0.001	3.1	0.015

NAP metabolites	Quartile 1	Ref		Ref		Ref	
	Quartile 2	2.34	0.013	2.14	0.037	2.01	0.143
	Quartile 3	2.09	0.030	1.99	0.060	3.02	0.022
	Quartile 4	3.48	<0.001	3.03	0.002	3.70	0.009

NAP metabolites; the sum of 1-hydroxynaphthalene and 2-hydroxynaphthalene (creatinine-corrected concentration (*μ*g/g creatinine)). ^∗^Models are adjusted for age, sex, mother's education, father's education, breastfeeding duration, passive smoking at home, formula feeding, breast milk, and consumption of cow's milk. ^∗∗^Models are adjusted for age, sex, mother's education, father's education, breastfeeding duration, passive smoking at home, formula feeding, breast milk and consumption of cow's milk, BMI, and 5 dietary patterns.

**Table 4:** Association of urinary levels of PAHs metabolites with type 1 diabetes in boys and girls.

Metabolites (*μ*g/g creatinine)		Crude models		Adjusted model^∗^		Adjusted model^∗∗^	
		OR	*P* value	OR	*P* value	OR	*P* value
Boys							
1-Hydroxynaphthalene	Quartile 1	Ref		Ref		Ref	
	Quartile 2	0.94	0.900	1.12	0.835	1.24	0.770
	Quartile 3	1.22	0.663	3.17	0.028	2.05	0.280
	Quartile 4	1.53	0.359	2.73	0.055	2.07	0.310

2-Hydroxynaphthalene	Quartile 1	Ref		Ref		Ref	
	Quartile 2	1.17	0.734	1.12	0.832	0.35	0.209
	Quartile 3	4.03	0.005	3.17	0.028	3.09	0.085
	Quartile 4	2.49	0.047	2.72	0.055	3.02	0.101

NAP metabolites	Quartile 1	Ref		Ref		Ref	
	Quartile 2	2	0.144	2.02	0.176	2.04	0.292
	Quartile 3	2.4	0.068	2.34	0.115	4.34	0.044^∗^
	Quartile 4	2.7	0.030	2.72	0.043	4.09	0.041^∗^

Girls							
1-Hydroxynaphthalene	Quartile 1	Ref		Ref		Ref	
	Quartile 2	0.74	0.548	0.84	0.752	1.39	0.727
	Quartile 3	1.25	0.642	1.56	0.419	3.40	0.173
	Quartile 4	1.74	0.252	1.81	0.277	2.08	0.437

2-Hydroxynaphthalene	Quartile 1	Ref		Ref		Ref	
	Quartile 2	3.57	0.019	4.22	0.024	10.6	0.032
	Quartile 3	6.4	0.001	5.81	0.004	6.21	0.044^∗^
	Quartile 4	7.5	<0.001	7.34	0.002	10.4	0.030^∗^

NAP metabolites	Quartile 1	Ref		Ref		Ref	
	Quartile 2	2.7	0.046	2.76	0.085	2.52	0.305
	Quartile 3	5.1	0.197	2.06	0.206	3.31	0.170
	Quartile 4	0.48	0.003	4.83	0.011	4.94	0.111

NAP metabolites; the sum of 1-hydroxynaphthalene and 2-hydroxynaphthalene (creatinine-corrected concentration (*μ*g/g creatinine)). ^∗^Models are adjusted for age, mother's education, father's education, breastfeeding duration, passive smoking at home, formula feeding, breast milk, and consumption of cow's milk. ^∗∗^Models are adjusted for age, mother's education, father's education, breastfeeding duration, passive smoking at home, formula feeding, breast milk and consumption of cow's milk, BMI, and 5 dietary patterns.

## Data Availability

The data that support the findings of this study are available on request from the corresponding author, (MH). The data are not publicly available due to the privacy of research participants.
